# Health related quality of life two to five years after gestational diabetes mellitus: cross-sectional comparative study in the ATLANTIC DIP cohort

**DOI:** 10.1186/s12884-015-0705-y

**Published:** 2015-10-24

**Authors:** Andriy Danyliv, Paddy Gillespie, Ciaran O’Neill, Eoin Noctor, Angela O’Dea, Marie Tierney, Brian E. McGuire, Liam G Glynn, Fidelma P Dunne

**Affiliations:** J.E. Cairnes School of Business and Economics, National University of Ireland, Galway, Ireland; School of Medicine, Clinical Sciences Institute, National University of Ireland, Galway, Ireland; School of Psychology, National University of Ireland, Galway, Ireland; Discipline of General Practice, National University of Ireland, Galway, Ireland; Galway Diabetes Research Centre, National University of Ireland Galway, Galway, Ireland

**Keywords:** Gestational diabetes mellitus, Health related quality of life, Post-partum, Health outcomes

## Abstract

**Background:**

There is no consensus on the effect of gestational diabetes mellitus (GDM) on health-related quality of life (HRQOL) for the mother in the short or long term. In this study we examined HRQOL in a group of women who had GDM in the index pregnancy 2 to 5 years previously and compared it to a group of women with normal glucose tolerance (NGT) in the index pregnancy during the same time period.

**Methods:**

The sample included 234 women who met International Association of Diabetes Study Groups (IADPSG) criteria for GDM in the index pregnancy and 108 who had NGT. The sample was drawn from the ATLATIC-DIP (Diabetes In Pregnancy) cohort – a network of antenatal centers along the Irish Atlantic seaboard serving a population of approximately 500,000 people. HRQOL was measured using the visual analogue component of the EQ-5D-3 L instrument in a cross-sectional survey.

**Results:**

The difference in HRQOL between GDM and NGT groups was not significant when adjusted for the effects of the covariates. HRQOL was negatively affected by increased BMI and abnormal glucose tolerance post-partum in the NGT group. Moderate alcohol consumption was positively associated with HRQOL in the NGT group only. The negative association with smoking on HRQOL was substantially higher in the GDM group.

**Conclusions:**

A diagnosis of GDM does not appear to have an adverse effect on HRQOL, 2 to 5 years after the index pregnancy. On the contrary, its diagnosis might lead to the development of coping strategies, which, consequently attenuates the adverse effect of the subsequent acquisition of abnormal glucose tolerance post-partum on HRQOL. Women whose pregnancy was affected by GDM are more susceptible to the adverse effects on HRQOL of alcohol use and tobacco smoking.

**Electronic supplementary material:**

The online version of this article (doi:10.1186/s12884-015-0705-y) contains supplementary material, which is available to authorized users.

## Background

Gestational diabetes mellitus (GDM) is defined as any degree of glucose intolerance with onset or first recognition during pregnancy and is associated with several maternal and neonatal complications [1]. Estimates of the prevalence of GDM vary across countries and ethnic groups, in part due to differences in diagnostic criteria [2]. However, there is a broad recognition that the prevalence is increasing primarily due to rising obesity levels [1, 3]. In the Irish population, GDM was estimated to affect 12.4 % of the pregnancies when universal screening and IADPSG criteria were used [4].

There is a large body of evidence showing the adverse consequences of GDM in the index pregnancy for both the mother and her offspring [4–6]. As a result, strategies to effectively screen and treat GDM have been implemented in many countries. A number of studies have shown that these strategies are associated with a reduction in perinatal complications [7, 8]. The National Institute for Health and Care Excellence (NICE) in the United Kingdom recognizes these strategies as being potentially cost-effective, though internationally evidence on their effectiveness is lacking [9].

For the purpose of resource allocation, a generic measure of effect, i.e. health-related quality of life (HRQOL), is of particular interest as it captures a more complex set of outcomes beyond clinical effects. Despite the growing body of knowledge on the detrimental clinical effects of GDM for mothers and infants, there is no consensus about its impact on HRQOL. A number of studies demonstrate that GDM is not associated with a reduced HRQOL [10, 11], lower perceived health status [12], or mood profile [13, 14], and does not cause depression or anxiety [11, 15]. By contrast, other studies demonstrate that GDM adversely affects HRQOL [16], perceived health status [17] and causes depression during pregnancy [18, 19] or after pregnancy [17]. In the long term, Feig et al., [20] reported that women affected by GDM in Canada report lower HRQOL (SF-36) 2 to 5 years after delivery. The authors [20] attribute this to the ‘labeling effect’ of the diagnosis rather than to real health consequences of GDM.

In this study, we add to the current literature by examining HRQOL 2 to 5 years post pregnancy in a sample of women who were part of the ATLANTIC-DIP (Diabetes in Pregnancy) cohort. This time frame allows for the capture of the long term effects of GDM [20]. Notably, the sample included two categories of women: (i) those diagnosed with GDM on IADPSG criteria following universal screening and (ii) those with normal glucose tolerance (NGT). In our analysis, we compared self-reported HRQOL, measured using the visual analogue scale (VAS) component of the EQ-5D-3 L in the two groups. Furthermore, we explored the patient characteristics (outlined in Appendix 1) that might also influence self-reported HRQOL in both patient cohorts.

## Methods

### Data

The study is based on the data collected within the framework of the ATLANTIC-DIP collaborative which has been described previously [4]. In brief, ATLANTIC-DIP is a network of antenatal centers along the Irish Atlantic seaboard serving a population of approximately 500,000 people. This regional area can be considered as broadly representative of the whole population of Ireland [21]. Pregnant women were offered screening at 24–28 weeks’ gestation using a 75-g oral glucose tolerance test (OGTT) with fasting, 1-h, and 2-h values. In total, 5,500 completed the screening. Henceforth, we refer to the pregnancy in this period as an index pregnancy. Of those screened, 12.4 % had GDM by IADPSG criteria [4].

In a subsequent follow-up study [22], 270 women with GDM (GDM group) and 388 women with NGT (NGT group) in the index pregnancy returned for a follow-up screen using a 75-g OGTT 2 to 5 years after the index pregnancy. In the current study, we build on this follow-up study by administering a postal questionnaire (in March 2013) to all of these women. This questionnaire contained questions on HRQOL assessed via the EQ-5D-3 L (with VAS component), healthcare service use, and socio-economic characteristics. The VAS component valued the HRQOL on a scale anchored from 0 (the worst health one can imagine) to 100 (the best health one can imagine). In total, 342 women responded to the questionnaire (52 % of the original sample) and were included in the analysis: 231 of these women had NGT and 111 had GDM in the index pregnancy. The participants of the study gave informed consent in writing at every stage of the study, i.e. initial and follow-up screening, and follow-up survey. The study was approved by the Irish Health Service Executive Clinical Research Ethics Committee (reference C.A.537).

### Outcomes

HRQOL was assessed via patient responses to EQ-5D-3 L. This is a multi-attribute instrument to measure HRQOL based on five health dimensions: mobility, self-care, usual activities, pain and discomfort, and anxiety and depression [23]. It also contains an instrument for a direct measurement of the self-reported HRQOL via VAS. In this study, we focus on the self-reported HRQOL via VAS only. The multi-attribute generic instrument EQ-5D-3 L produced highly homogenous results which in general exceeded the self-reported status (see Additional file 1). Hence, we found this generic instrument to be insensitive for detecting HRQOL differences in women affected by GDM.

Analysis showed that the HRQOL measurement had a skewed distribution which could not be corrected by logarithmic transformation. Therefore, the utility decrement (henceforth UD), representing the distance to perfect health was applied. The UD is measured as:

1 $$ UD=100- HRQOL $$

The distribution of UD was still found to significantly differ from the normal based on a Shapiro-Wilk test, as was the HRQOL. However, its properties (unbounded on logarithmic scale) preconditioned its use for further analysis in the regression models.

### Covariates

For the multivariate analysis, a set of covariates were chosen to investigate the impact of a diagnosis of GDM on HRQOL. Selection of covariates was based in part on previous studies [10] and in part on that which was available in the dataset. The detailed definition of the variables may be found in Additional file 2. Briefly, the main groups of factors were the indication of acquired abnormal glucose tolerance after the pregnancy, age, life-style indicators (BMI, fruit and vegetable intake, exercising 30 min a day), risky behavior in terms of alcohol and tobacco use, mode of delivery in the index pregnancy, subsequent miscarriages, and a set of socio-economic indicators (income, employment and cohabiting status). The two indicators, possession of a ‘medical card’ and private health insurance, were introduced in the analysis as they might have impacted on health service utilization, and consequently on HRQOL, and may represent socio-economic status as well [24, 25]. In Ireland, a medical card is issued based on low income, age, or financial hardship as a result of a medical condition and entitles the holder to free health care. Private health insurance, depending on the level chosen, can cover some health care costs and is usually purchased by people with high income. We also controlled for the time elapsed after the index delivery before the HRQOL measurement.

### Statistical analysis

A series of univariate analyses were undertaken to examine if the samples differed by the characteristics mentioned above. This consisted of independent two-tailed t-tests for continuous variables, and χ^2^ tests for categorical variables. For the multivariate analyses, the choice of estimation approach was informed by the nature of the dependent variable, i.e. utility decrement. Specifically, a generalized linear model with log link and Gaussian family of the error term distribution was selected (the distribution of the utility decrement is intrinsically continuous and, therefore, Gaussian family was preferred, although Modified Park’s test favored Poisson’s distribution). The coefficients in the model represent the effect on the utility decrement on the logarithmic scale and, therefore, have opposite effect on the HRQOL, i.e. positive coefficients mean reduction in HRQOL and negative – increase in HRQOL.

The women in our study were selected from a number of antenatal study centers. In order to detect explicitly if there were differences between the study centers, we applied center-specific constant effects. Statistical significance was explored at three levels (0.05, 0.01, and 0.001). All analyses were performed using STATA 12 software.

## Results

### Summary statistics

The descriptive statistics for the sample are presented in Table 1. The two groups appear to be different in many of the presented characteristics. Most importantly, the group of GDM women tended to have slightly lower HRQOL than the group with NGT, i.e. 80.31 *vs* 83.98. As expected, GDM women appeared worse off in terms of health outcomes and health related behavior, i.e. they more frequently acquired abnormal glucose tolerance post-partum, were more prone to be overweight or obese, to have a caesarian section delivery. There was some imbalance between the groups in terms of distribution across study centers, but in the multivariate analysis, study center effect was controlled for. Notably, GDM women were less likely to consume alcohol, though this difference was at the verge of significance. In our case, alcohol consumption represents moderate alcohol consumption as high consumption (14–18 units per week) was reported only in 3 cases.

**Table 1:** Descriptive statistics of the study sample

		NGT		GDM		*p*-value
		N	Statistics	N	Statistics	
Total sample size		231		111		
HRQOL(VAS), score 0-100	mean (std.err.)	231	83.98 (0.79)	111	80.31 (1.36)	0.020
Age, years	mean (std.err.)	231	38.99 (0.31)	111	38.15 (0.44)	0.120
Alcohol cons., units/week	mean (std.err.)	231	2.39 (0.20)	107	1.97 (0.28)	0.232
Income (midpoint), €	mean (std.err.)	202	36,139 (1044)	91	37,198 (1584)	0.577
Abnormal glucose tolerance post-partum						
no	%	224	97.0 %	92	82.9 %	<0.001
yes	%	7	3.0 %	19	17.1 %	
Time after delivery						
year 2	%	5	2.2 %	32	28.8 %	<0.001
year 3	%	20	8.7 %	23	20.7 %	
year 4	%	103	44.6 %	33	29.7 %	
year 5	%	103	44.6 %	23	20.7 %	
Alcohol consumption						
no	%	78	33.8 %	47	43.9 %	0.072
yes	%	153	66.2 %	60	56.1 %	
missing	%	0		4		
Smoking status						
past	%	70	30.3 %	43	38.7 %	0.283
current	%	30	13.0 %	14	12.6 %	
never	%	131	56.7 %	54	48.6 %	
BMI (3 classes)						
normal or underweight	%	120	51.9 %	35	31.8 %	<0.001
overweight	%	80	34.6 %	42	38.2 %	
obese	%	31	13.4 %	33	30.0 %	
missing	%	0		1		
Subsequent miscarriages						
no	%	196	90.7 %	97	88.2 %	0.469
yes	%	20	9.3 %	13	11.8 %	
missing	%	15		1		
Mode of delivery						
spontaneous vaginal	%	142	63.1 %	47	42.3 %	<0.001
caesarian section	%	52	23.1 %	52	46.8 %	
assisted vaginal	%	31	13.8 %	12	10.8 %	
missing	%	6		0		
Cohabiting status						
single	%	20	8.7 %	11	9.9 %	0.715
cohabiting	%	210	91.3 %	100	90.1 %	
missing	%	1		0		
Employment status						
not employed	%	84	36.7 %	39	35.1 %	0.781
employed or self-empl.	%	145	63.3 %	72	64.9 %	
missing	%	2		0		
Household income level (€)						
<10,000	%	10	5.0 %	1	1.1 %	0.184
10,0000-19,999	%	20	9.9 %	15	16.5 %	
20,000-29,999	%	45	22.3 %	17	18.7 %	
30,000-39,999	%	35	17.3 %	15	16.5 %	
40,0000-49,0000	%	46	22.8 %	16	17.6 %	
50,000+	%	46	22.8 %	27	29.7 %	
missing	%	29		20		
Medical card						
no	%	145	63.3 %	69	62.2 %	0.836
yes	%	84	36.7 %	42	37.8 %	
missing	%	2		0		
Private health insurance						
no	%	88	39.1 %	49	44.1 %	0.377
yes	%	137	60.9 %	62	55.9 %	
missing	%	6		0		
Daily fruit and veg. intake						
no	%	5	2.2 %	6	5.4 %	0.112
yes	%	226	97.8 %	105	94.6 %	
Exercise 30 min a day						
no	%	34	14.7 %	10	9.0 %	0.140
yes	%	197	85.3 %	101	91.0 %	
Study center						
Site A	%	130	56.3 %	40	36.0 %	<0.001
Site B	%	40	17.3 %	9	8.1 %	
Site C	%	38	16.5 %	32	28.8 %	
Site D	%	23	10.0 %	30	27.0 %	

A potential problem is the small numbers in subgroups of some variables (acquired abnormal glucose tolerance, regular fruit and vegetable consumption). Another potential problem is subgroups with missing values for income and subsequent miscarriages. Full case analysis (excluding cases with missing value in at least one of the covariates) might cause loss of information. Therefore, the full case multivariate models of the utility decrement were compared to the models where missing values were included as a separate category. As the results did not differ substantially between the two specifications, we present the full case analysis below.

### HRQOL patterns

The results of the multivariate analysis of the utility decrement for the GDM and the NGT groups, and the pooled estimates are presented in Table 2. In general, the models fit well. The center-specific effects are not significant and their exclusion improves model fit, which tells us that there is no heterogeneity of HRQOL across the study centers (no evidence of selection bias by the study center). Despite the statistically significant difference between mean observed self-reported HRQOL in GDM and NGT groups (unpaired *t*-test), the results of the pooled multivariate analysis (accounting for other covariates) demonstrate that GDM per se does not have a long term effect on HRQOL. There are, however, differences in the effect of other covariates between GDM and NGT groups.

**Table 2:** Results of the GLM regression of the utility decrement (log link, Gaussian family)

		Pooled	NGT	GDM
		*coeff. (std.err)*	*coeff. (std.err)*	*coeff. (std.err)*
GDM	Yes (positive)	0.047		
		(0.105)		
Abnormal glucose tolerance	Yes (positive)	0.251	0.566*	0.125
		(0.152)	(0.254)	(0.247)
Time after delivery	2 years = ref.			
	3 years	0.461*	−0.238	0.475
		(0.180)	(0.335)	(0.258)
	4 years	0.182	−0.603*	0.200
		(0.167)	(0.280)	(0.244)
	5 years	0.063	−0.565*	0.088
		(0.171)	(0.277)	(0.248)
Alcohol consumption	Yes	−0.279**	−0.292*	−0.294
		(0.095)	(0.135)	(0.198)
Smoking status	Past smoker	0.078	0.048	0.440*
		(0.112)	(0.159)	(0.255)
	Current smoker	0.504***	0.415**	0.823**
		(0.125)	(0.160)	(0.261)
	Never sm. = ref.			
BMI classification	Norm./Underweight = ref.			
	Overweight	0.159	0.259*	0.168
		(0.115)	(0.130)	(0.238)
	Obese	0.436***	0.513**	0.341
		(0.130)	(0.177)	(0.238)
Age		0.001	0.013	−0.004
		(0.012)	(0.014)	(0.023)
Had subsequent miscarriages	Yes	0.235*	−0.015	0.135
		(0.128)	(0.212)	(0.239)
Mode of delivery	SVD	−0.033	−0.116	−0.008
		(0.109)	(0.136)	(0.223)
	CS = ref.			
	Assisted	−0.011	−0.023	−0.261
		(0.163)	(0.185)	(0.401)
Cohabiting status	cohabits = 1	0.244	0.535	−0.004
		(0.164)	(0.301)	(0.327)
Employment status	employed = 1	0.021	0.312*	−0.129
		(0.102)	(0.146)	(0.187)
Income level	<20,000 = ref.			
	20,000-29,999	−0.383*	−0.492**	−0.132
		(0.156)	(0.188)	(0.386)
	30,000-39,999	−0.096	−0.259	0.046
		(0.158)	(0.170)	(0.466)
	40,0000-49,0000	−0.292	−0.749***	0.372
		(0.158)	(0.199)	(0.380)
	50,000+	−0.319	−0.637**	0.087
		(0.167)	(0.203)	(0.400)
Medical card	Yes	−0.067	−0.098	−0.011
		(0.139)	(0.171)	(0.241)
Private health insurance	Yes	−0.008	0.003	0.064
		(0.125)	(0.146)	(0.219)
Fruit and veg. consumption	Yes	−0.157	−0.174	−0.650
		(0.261)	(0.408)	(0.413)
Exercises 30 min a day	Yes	−0.116	−0.099	−0.229
		(0.139)	(0.163)	(0.323)
Center effect	Site A = ref.			
	Site B	0.153	0.165	0.237
		(0.135)	(0.177)	(0.322)
	Site C	−0.149	−0.063	−0.415*
		(0.140)	(0.180)	(0.250)
	Site D	0.174	−0.078	0.154
		(0.131)	(0.241)	(0.220)
Constant		2.760***	2.773***	3.350**
		(0.571)	(0.740)	(1.037)
*N*		261	175	86
Log-likelihood		−1,008.31	−657.09	−329.37
P-value (Log-likelihood test)		<0.001	<0.001	0.04

*p < 0.05; **p < 0.01; ***p < 0.001

The coefficients of the income effect have signs consistent with intuition in the NGT group, i.e. higher income is associated with higher HRQOL (lower utility decrement in the model). The HRQOL in the GDM group appears not to be affected by any indicators of socio-economic status, while income and employment status are significant factors in the NGT group.

A similar pattern is observed for BMI and development of abnormal glucose tolerance post-partum. Post pregnancy abnormal glucose tolerance is associated with a significant reduction in HRQOL in the NGT group. In the GDM group, this effect is not significant. In the NGT group, HRQOL is lower for those in the overweight group (BMI 25–30) and even lower for those classified as obese (BMI ≥ 30), while in the GDM group, their HRQOL is not statistically different from that of women with normal or reduced body mass (BMI <25).

Alcohol and tobacco use appears to have more adverse effects in the GDM group. Moderate alcohol consumption is associated with better HRQOL only in the NGT group. Smoking (current smoking) is associated with reduced HRQOL in both groups, but the effect is much stronger in the GDM group.

We found no relation between HRQOL and the indicators of social status and health service access, i.e. cohabiting status, medical card, and private health insurance in any of the study groups. Notably, no association was found between HRQOL and age, which may be explained by homogeneity between groups on this characteristic (age ranges 24 to 51 with low variation). Similarly, mode of delivery, subsequent miscarriages, or lifestyle indicators (fruit and vegetable consumption and exercising 30 min a day) were not associated with the HRQOL level.

## Discussion

In this study we compared self-reported HRQOL for GDM and NGT women 2 to 5 years post pregnancy. We also explored patient characteristics which may influence self-reported HRQOL in both patient cohorts. Below, we discuss the main findings of this study and acknowledge the study limitations.

### GDM effect

Our results suggest that GDM or its diagnosis per se does not a have substantial adverse effect on HRQOL 2 to 5 years after the index pregnancy. Even though women with GDM tended to have slightly lower reported HRQOL than those who had NGT (80.3 versus 84.0), this difference is not statistically significant if other clinical and socio-economic factors are accounted for. Thus, we are discussing the independent association of GDM diagnosis with HRQOL, adjusted for the associations caused by other factors in the regression. Our results add to the evidence that GDM does not have an effect on HRQOL [10, 11]. We consider this to be a counter-argument to the studies suggesting adverse ‘labeling effect’ of a GDM diagnosis on the wellbeing of a woman [20]. Even if such an effect is present, it is negligible. It should be borne in mind, however, that these results are estimated from data collected in a region where GDM diagnosis leads to an appropriate treatment, i.e. lifestyle advice, blood glucose self-monitoring, and if required, insulin. They might not hold for other institutional and cultural contexts, especially where GDM is not diagnosed and/or treated in a similar fashion.

### Diabetes and abnormal glucose tolerance

Notably, after a GDM pregnancy, women’s HRQOL appears not to be affected by acquisition of abnormal glucose tolerance or diabetes post-partum, while in the group of NGT women it appeared to have a negative effect. Previous evidence demonstrates that people with diabetes tend to have lower HRQOL than people without diabetes, but proper management of this condition eliminates this reduction [13, 26]. Thus, it might be hypothesized that women affected by GDM are likely to develop coping strategies or to be offered services in response to GDM. That is, they undergo certain treatment procedures and acquire management skills which enable them to be better able to manage and control abnormal glucose tolerance upon its development post pregnancy. In contrast, women with NGT at pregnancy may be less well equipped with such strategies upon development of abnormal glucose tolerance post-partum; hence, their HRQOL appears reduced after the diagnosis.

### Risky behavior

Our results indicate that women whose pregnancy was affected by GDM are more susceptible to the adverse effects of risky behavior, alcohol use and tobacco smoking in particular, 2 to 5 years after the affected delivery. We draw this conclusion based on the observation that current smoking status has a much stronger negative effect on HRQOL in the GDM women, while moderate alcohol consumption is favorable for the HRQOL of women who had NGT in the index pregnancy. The cross-sectional design of our study does not allow us to explore the mechanisms of these associations; however, these findings are consistent with the clinical literature. Smoking might facilitate progression to Type 2 diabetes mellitus (DM) after GDM [27] and is associated with excessive negative health outcomes in people with diabetes due to circulatory and cardiovascular complications [28]. There is no clear link between alcohol consumption and GDM and its effects. However, in people with diabetes, which is more likely to develop in women with prior GDM [29], alcohol use worsens glucose control and might hasten complications [30]. The exact mechanisms of alcohol and tobacco use in relation to HRQOL and its components after GDM might be interrogated in a more detailed cohort study. Our results suggest increased attention should be given to alcohol and tobacco consumption after a GDM pregnancy.

### Limitations of the study

It should be noted that data collection for this study was powered to answer the question about transition from GDM to abnormal glucose tolerance or Type 2 DM rather than to study heterogeneity of HRQOL in the two groups. There was a 48 % non-response rate and GDM women were more likely to be non-respondents (59 %) compared to NGT women (41 %). Moreover, our estimates of post-partum abnormal glucose tolerance status are lower than those in the original sample of the women who had a follow-up screening, i.e. 3.0 % *vs* 3.6 % in the NGT group, and 17.1 % *vs* 26 % in the GDM group. Thus, there might be some non-response bias embedded in the current study as the non-respondents seem to have worse health status.

Some relations should be considered cautiously due to the small size of the subgroups. Similarly, significant relationships could have been undetected for the same reason. Additionally, small subgroups did not allow for the inclusion of the interactions between the factors.

Another limitation of this study is the overrepresentation of the GDM group. In this study, it makes up 1/3 of the total sample, while GDM happens in about 12 % of all pregnancies [4]. However, substantial group sizes of GDM and NGT women allow for studying the HRQOL heterogeneity separately in these groups.

Finally, we focus on the self-reported HRQOL via visual analogue scale only as the generic EQ-5D-3 L appeared to be insensitive in our sample. Application of the diabetes-specific HRQOL tools might yield different results and better detect the differences in HRQOL attributed to diabetic states.

## Conclusions

Our results suggest that GDM diagnosis per se does not have an adverse effect on HRQOL 2 to 5 years after the index pregnancy. On the contrary, its diagnosis might lead to the development of coping strategies, i.e. proper treatment and monitoring of the glucose level, which consequently may attenuate the adverse effect of the post-partum development of abnormal glucose tolerance on HRQOL. Alcohol use and smoking appear to have worse effects on HRQOL in women with prior GDM compared to NGT controls. However, the mechanisms of these effects are not clear and merit further exploration.

## Additional files

Additional file 1: Table S1. The descriptive statistics of the HRQOL assessed via EQ-5D-3 L and its components and its difference between NGT and GDM groups. (DOC 57 kb) Additional file 2:
**Definition of the explanatory variables.** (DOC 27 kb)

## Abbreviations

ATLANTIC DIP: Atlantic, Diabetes in Pregnancy
BMI: Body mass index
DM: Diabetes Mellitus
GDM: Gestational Diabetes Mellitus
HRQOL: Health related Quality of Life
NGT: Normal glucose tolerance
UD: Utility decrement
VAS: Visual analogue scale
