# Supplementary material for: Health related quality of life two to five years after gestational diabetes mellitus: cross-sectional comparative study in the ATLANTIC DIP cohort
Source: BMC Pregnancy Childbirth. 2015 Oct 24;15:274. doi: 10.1186/s12884-015-0705-y (PMC4619994; doi:10.1186/s12884-015-0705-y)
Supplement: Additional file 1: Table S1. — The descriptive statistics of the HRQOL assessed via EQ-5D-3 L and its components and its difference between NGT and GDM groups. (DOC 57 kb) [file 12884_2015_705_MOESM1_ESM.doc]

**Additional file 1**

**Table AF2.1 - The descriptive statistics of the HRQOL assessed via EQ-5D-3L and its components and its difference between NGT and GDM groups**

|  |  | **NGT** | | **GDM** | | **P-value** |
| --- | --- | --- | --- | --- | --- | --- |
|  |  | **N** | **Statistics** | **N** | **Statistics** |
| **Total sample size** | | **231** |  | **111** |  |  |
| **EQ-5D-3L,** score 0-100: mean (std.err.) | | 228 | 93.6 (0.82) | 108 | 94.0 (1.13) | 0.814 |
| **Mobility** | |  |  |  |  |  |
|  | I have no problems in walking about | 222 | 96.5% | 108 | 98.2% | 0.397 |
|  | I have some problems in walking about | 8 | 3.5% | 2 | 1.8% |  |
|  | Missing | 1 |  | 1 |  |  |
| **Self-care** | |  |  |  |  |  |
|  | I have no problems with self-care | 230 | 100.0% | 109 | 100.0% | n.a. |
|  | Missing | 1 |  | 2 |  |  |
| **Usual Activities** | |  |  |  |  |  |
|  | I have no problem with performing my usual activities | 219 | 95.2% | 106 | 95.5% | 0.909 |
|  | I have some problems with performing my usual activities | 11 | 4.8% | 5 | 4.5% |  |
|  | Missing | 1 |  | 0 |  |  |
| **Pain and Discomfort** | |  |  |  |  |  |
|  | I have no pain or discomfort | 186 | 80.9% | 89 | 80.9% | 0.999 |
|  | I have moderate pain or discomfort | 42 | 18.3% | 20 | 18.2% |  |
|  | I have extreme pain or discomfort | 2 | 0.9% | 1 | 0.9% |  |
|  | Missing | 1 |  | 1 |  |  |
| **Anxiety and Depression** | |  |  |  |  |  |
|  | I am not anxious or depressed | 196 | 85.2% | 94 | 84.7% | 0.353 |
|  | I am moderately anxious or depressed | 34 | 14.8% | 16 | 14.4% |  |
|  | I am extremely anxious or depressed | 0 | 0.0% | 1 | 0.9% |  |
|  | Missing | 1 |  | 0 |  |  |

*n.a. – not applicable*

Table S2.1 demonstrates the summary of the integral EQ-5D-3L score obtained by assigning UK-specific weights to the 5 dimensions applied in this instrument in the NGT and GDM groups. The t-test is applied to test the difference in this score between the two groups. The second part of the Table S2.1 also provides the distribution of the valuation of the 5 dimensions of the EQ-5D-3L instrument and the analysis of the association with the GDM status via χ^2^ test.

In summary, there is no detectable difference between the NGT and GDM groups in the integral EQ-5D-3L score or its components. However, such picture might occur not only due to true absence of the differences, but also due to low sensitivity of the generic EQ-5D-3L instrument to the problems reducing HRQOL in GDM. High homogeneity of the EQ-5D-3L scores and absence of detectable differences preconditioned the use of Visual Analogue Scale (VAS) scores which demonstrated higher variability within and across the study groups. The summary statistics and the analysis of the VAS scores can be found in the main text of the paper.
