# Supplementary material for: Health related quality of life two to five years after gestational diabetes mellitus: cross-sectional comparative study in the ATLANTIC DIP cohort
Source: BMC Pregnancy Childbirth. 2015 Oct 24;15:274. doi: 10.1186/s12884-015-0705-y (PMC4619994; doi:10.1186/s12884-015-0705-y)
Supplement: Additional file 2: — Definition of the explanatory variables. (DOC 27 kb) [file 12884_2015_705_MOESM2_ESM.doc]

**Additional file 2**

**Definition of the explanatory variables**

- **Abnormal glucose tolerance** is a binary indicator taking value 1 if a woman was diagnosed with abnormal glucose tolerance by IADPSG criterion either post-partum or 2 to 5 years after the delivery, and 0 if she had normal glucose tolerance at both readings.
- **Time after delivery** measured in full years elapsed after the index delivery when a woman was tested for GDM before the HRQOL measurement.
- **Alcohol consumption** is a binary indicator taking value 1 if a woman reports any consumption of alcohol units per week, and 0 if no consumption. Only 3 cases reported consumption of more than 14 units per week with maximum reported 18 units. Thus, this indicator is identical to the indication of moderate alcohol consumption versus no consumption.
- **Smoking status** is a nominal variable indicating one of the three mutually exclusive categories: “past smoker”, “current smoker”, and “never smoked”.
- **BMI (mass/height^2^)**, calculated at the first obstetrical visit, was used to generate a three-category variable for the analysis: normal or reduced weight was classified as a BMI of less than 25 kg/m2, overweight as between 25 and 30 kg/m2, and obese as a BMI exceeding 30 kg/m2.
- **Age** was included as a continuous characteristic. It was also checked for the impact on HRQOL in categories (possibly non-monotonous relation to HRQOL), but none of the categories displayed significantly different HRQOL level.
- The indicator of **subsequent miscarriages** indicates whether a woman had miscarriage in the period between the index pregnancy when she was tested for GDM and HRQOL measurement (value 1) or not (value 0).
- **Mode of delivery** indicates whether the index pregnancy ended in spontaneous vaginal delivery (SVD), caesarian section (CS), or assisted delivery (Assisted).
- **Cohabiting status** is a binary indicator taking value 1 if a woman reported cohabiting with a partner or a spouse, or 0 if she reported to be single.
- **Employment status** is a binary indicator taking value 1 if a woman reported being employed or self-employed, and 0 if she reported to be unemployed or managing household.
- **Income level** represents combined household annual income from all sources and was measured at the time of last clinical readings (less than a year to HRQOL measurement) via income intervals with a € 10’000 step. Due to very small frequency in the first interval (<€ 10’000) it was combined with the second interval resulting in the following mutually exclusive ordered groups: “less than € 20’000”, “€ 20’000 - € 29’999”, “€ 30’000 - € 39’999”, “€ 40’000 - € 49’999”, “€ 50’000 and more”.
- **Medical card** is a binary indicator taking value 1 if a woman reported possessing a medical card and 0 if she reported to not having it.
- **Private health insurance** is a binary indicator taking value 1 if a woman reported possessing a private health insurance policy and 0 if she reported to not having it.
- **Fruit and vegetable consumption** is a binary indicator taking value 1 if a woman reported consuming fruit and vegetables regularly every day and 0 otherwise.
- **Exercise** is a binary indicator taking value 1 if a woman reported exercising regularly at least 30 minutes a day and 0 otherwise.
- **Center effect** indicated one of the four study centers where women were recruited and followed up. This included: Galway, Portiuncula, Mayo, or Letterkenny.
